# Malignant Epithelial Transformation in Phyllodes Tumor: A Population-Based Case Series

**DOI:** 10.7759/cureus.1815

**Published:** 2017-11-03

**Authors:** Ralph L Widya, Myra F Rodrigues, Pauline T Truong, Peter H Watson, Lorna M Weir, Margaret A Knowling, Elaine S Wai

**Affiliations:** 1 Radiology, Leiden University Medical Center; 2 Radiation Oncology, Erasmus Mc Cancer Institute; 3 Radiation Oncology, University of British Columbia, BC Cancer Agency; 4 Pathology, University of British Columbia, BC Cancer Agency; 5 Medical Oncology, University of British Columbia, BC Cancer Agency

**Keywords:** phyllodes tumor, breast, invasive ductal carcinoma, ductal carcinoma in situ, lobular carcinoma in situ, radiation oncology, pathology, oncology

## Abstract

Background

Phyllodes tumor (PT) of the breast is an uncommon fibroepithelial neoplasm. Malignant epithelial transformation in PT is rare. This study reports clinicopathologic characteristics and outcomes of patients with malignant epithelial transformation in PT.

Methods

From an institutional database of 183 patients with newly diagnosed PT referred to a Canadian provincial cancer institution between 1999 and 2014, 11 cases of PT with concomitant in situ or invasive carcinoma were identified. Descriptive analysis was performed to document the characteristics, treatment and outcomes of this cohort.

Results

Prevalence of malignant epithelial transformation in PT was 6.0%. Median (range) age was 54 (35–75) years. Types of carcinoma were ductal carcinoma in situ (DCIS) (n = 6), lobular carcinoma in situ (n = 4), and invasive ductal carcinoma (IDC) (n = 1). Median PT size was 5 (1–15) cm. Three PTs were classified as benign (27%), five as borderline (45%), and three as malignant (27%). Mastectomy was performed in six (55%) and breast conserving surgery in five (45%) patients. Hormonal therapy was used in two cases: one with a 1 cm, grade 2 DCIS, and one with an 11 cm, grade 1 IDC, the latter also receiving radiotherapy. Mean follow-up duration was 54 (6–175) months. None of the cases showed any evidence of disease after treatment at the time of their last follow-up.

Conclusion

This case series showed a higher prevalence of malignant epithelial transformation in PT than reported in previous literature. Outcomes were favourable despite the presence of either in situ or invasive carcinoma within PT.

## Introduction

Phyllodes tumor (PT) of the breast is an uncommon fibroepithelial neoplasm with proliferation of both epithelial and stromal components, accounting for less than 1% of all breast tumors. PTs are classified as benign, borderline or malignant according to histologic features defined by the World Health Organization (WHO) [1]. Malignancy is typically found in the stromal component, whereas malignant epithelial transformation in PT is rare. A study with a large series of women with PT reported that the prevalence of carcinoma arising within PT was <1% [2]. Since the first case report of carcinoma associated with PT in 1954 [3], several other case reports have been published. Some studies included reviews of the literature with up to 39 individual cases [4-8]. To date, only two case series of patients with in situ or invasive carcinoma coexisting with PT have been published [9, 10]. These case series included patients with carcinoma within PT, as well as carcinoma occurring in the same breast but separate from the PT.

The aim of this population-based series was to investigate the prevalence of malignant epithelial transformation in patients with newly diagnosed PT, and to evaluate clinicopathologic features, management and outcome in individual cases.

## Materials and methods

The British Columbia Cancer Agency (BCCA) is a tertiary Canadian cancer care institution providing services throughout the province of British Columbia. The institutional database of the BCCA was reviewed and all cases with newly diagnosed PT who were referred to the BCCA for management between January 1, 1999 and December 31, 2014 were extracted. Pathology reports were evaluated. Histological diagnoses were made at final specimen evaluation. Cases were included when malignant epithelial transformation in PT was reported. Patient demographics, history of breast disease, histological features, treatment and follow-up were examined. The study was approved by the University of British Columbia-BCCA Research Ethics Board.

## Results

Data of 183 patients with newly diagnosed PT were extracted from the BCCA institutional database. Malignant epithelial transformation was present in 11 cases (6.0%, all female). Central pathology review by the BCCA was performed in eight of these 11 cases (73%). The proportion of central pathology review was comparable to the proportion of the entire cohort (71%). Median age at diagnosis was 54 years (range: 35–75 years). Table 1 shows the clinicopathologic characteristics of all cases. Pathology reports were available for all patients, and secondary review of histology slides at the BCCA was performed in eight cases. Types of carcinoma were: ductal carcinoma in situ (DCIS) (n = 6), lobular carcinoma in situ (n = 4), and invasive ductal carcinoma (IDC) (n = 1). Median PT size was 5 cm (range: 1–15 cm). Three PTs were nevertheless classified on the basis of the stromal component as benign (27%), five as borderline (45%), and three as malignant (27%) histology. Histological features of the phyllodes tumors are shown in Table 2. All patients presented with local breast disease and with palpable tumor on clinical examination.

**Table 1:** Clinicopathologic characteristics. PT: Phyllodes tumor; LCIS: Lobular carcinoma in situ; NA: Not available; FA: Fibroadenoma; BCS: Breast conserving surgery; DCIS: Ductal carcinoma in situ; NOS: Not otherwise specified; IDC: Invasive ductal carcinoma. * Fine needle aspiration cytology: suspicious for malignancy.

Case no.	Age (years)	Menopausal status	History of breast disease	Core needle biopsy histology	Definitive histology PT subtype	PT size (cm)	PT margin status	Associated epithelial disease	Grade of carcinoma	Size of carcinoma (cm)	Type of surgery	Adjuvant therapy
1	42	Unknown	No	Benign PT	Borderline	15	Negative	LCIS	NA	Focal	Mastectomy	No
2	54	Post	FA	Benign PT	Benign	6	Negative	LCIS	NA	Extensive	BCS	No
3	75	Post	FA	Fibroepithelial lesion	Malignant	5	Negative	DCIS	1	0.2	Mastectomy	No
4	44	Pre	No	Fibroepithelial lesion	Benign	4	Close	DCIS	3	0.25	BCS	No
5	49	Unknown	No	Fibroepithelial lesion	Malignant	4	Negative	DCIS	1	4	Mastectomy	No
6	59	Post	No	Fibroepithelial lesion	Borderline	9	Negative	LCIS, DCIS	NA, 3	Focal, 1.1	Mastectomy	No
7	35	Pre	No	Fibroepithelial lesion	Benign	5	Close	LCIS	NA	Focal	BCS	No
8	69	Post	No	PT NOS	Borderline	4	Close	DCIS	1	Scattered foci	BCS	No
9	58	Post	FA	Fibroepithelial lesion	Borderline	11	Negative	IDC	1	11	Mastectomy, lymph node dissection	Radiotherapy, anastrozole
10	53	Pre	No	FA, DCIS, PT	Borderline	5	Negative	DCIS	2	Multiple foci 0.4-1	Mastectomy	Tamoxifen
11	53	Pre	No	Not performed*	Malignant	1	Negative	LCIS	NA	0.3	BCS	No

**Table 2:** Histological features of phyllodes tumor. HPF: High power fields; NA: Not available.

Case no.	Mitoses per 10 HPF	Cellular atypia	Stromal overgrowth	Necrosis	Stromal hypercellularity	Type of tumor borders
1	5-9	Yes	NA	No	NA	Intermediate
2	0-4	Yes	NA	No	Modest/moderate	Intermediate
3	≥10	Yes	Yes	No	Marked	Invasive
4	0-4	Yes	No	No	Modest/moderate	Pushing/circumscribed
5	≥10	Yes	Yes	No	Modest/moderate	Invasive
6	≥10	Yes	No	No	Modest/moderate	Intermediate
7	0-4	Yes	No	No	Modest/moderate	NA
8	0-4	Yes	NA	No	Modest/moderate	Pushing/circumscribed
9	0-4	Yes	NA	No	Modest/moderate	Intermediate
10	5-9	Yes	Yes	Yes	Marked	Pushing/circumscribed
11	5-9	Yes	Yes	No	Marked	Intermediate

**Figure 1:**
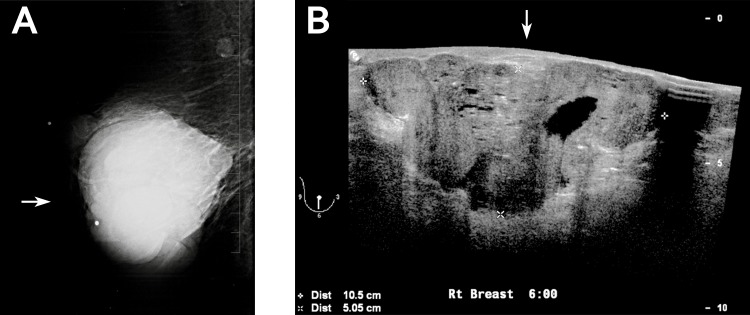
Case of invasive ductal carcinoma within a borderline phyllodes tumor. Mammogram (A) and ultrasound (B) of the right breast showed a large macrolobulated circumscribed solid mass without substantial vascularity. Microcystic spaces and pleomorphic microcalcifications were present within the mass.

**Figure 2:**
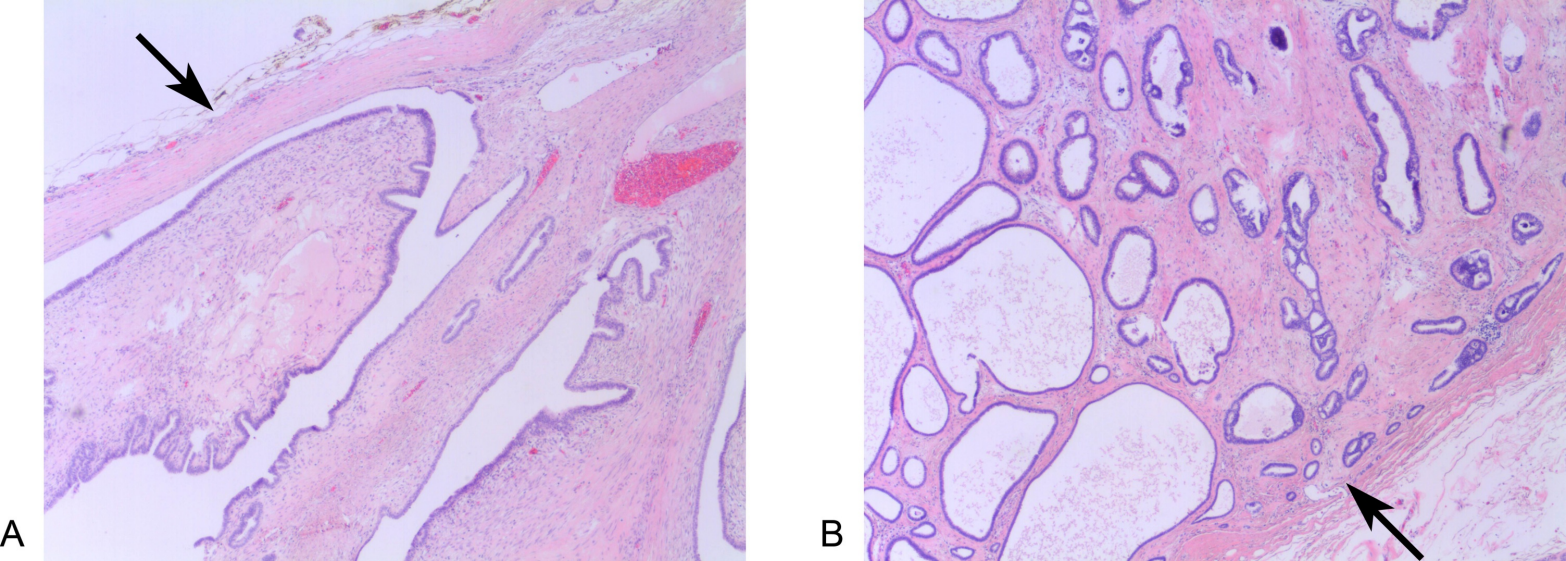
Histopathology of invasive ductal carcinoma within a borderline phyllodes tumor. Low power views (4x) of classical phyllodes tumor component (A) and of low grade invasive component (B).

Mastectomy was performed in six (55%) and breast conserving surgery in five (45%) patients. Three out of the six DCIS cases were low grade. One patient with multiple foci of grade 2 DCIS ranging from 4 to 10 mm, with estrogen receptors (ER) positive status, was treated with mastectomy and received adjuvant hormonal therapy (tamoxifen) for four years. One patient with grade 3 DCIS concomitant with PT was treated with breast-conserving surgery alone. In this case, surgical margins to the PT were close (0.5 mm), but because the focus of DCIS measured 2.5 mm and the margins to the in situ carcinoma were 2.5 cm, this patient did not receive adjuvant therapy. The second patient with grade 3 DCIS was treated with mastectomy with clear margins, and therefore did not receive adjuvant therapy. One patient was diagnosed with pathologic T3N0M0 IDC, which infiltrated throughout a borderline PT of 11 cm. This IDC was grade 1, ER positive, progesterone receptor-positive and Her2/neu-negative cancer without lymphovascular invasion (Figures 1, 2), treated with mastectomy and axillary dissection, followed by adjuvant locoregional radiotherapy (50.4 Gy in 28 fractions) and hormonal therapy (anastrozole) for 10 years. Mean follow-up duration was 53.8 months (range: 6–175 months). At the time of their last follow-up, all patients were alive and none of them showed any evidence of disease recurrence.

## Discussion

Reports on malignant epithelial transformation in PT mainly consist of anecdotal reports. We hereby add 11 cases to the current literature that were present in a population-based cohort of a tertiary oncology facility in British Columbia, Canada. To the best of our knowledge, this is the largest case series of patients with this rare combination of pathologies.

Breast cancer may coexist with PT in three situations: within the PT in the ipsilateral breast, or separate from the PT in either the ipsilateral or contralateral breast. Whereas etiology might be coincidental in carcinoma arising well away from the PT [11], the underlying etiology of carcinoma occurring within the PT is unknown. The current series comprised only patients with carcinoma within the PT. Genetic changes in both the epithelium and stroma of PTs have been described suggesting that both are part of the neoplastic process [12]. It is, however, unclear if the malignant transformation of epithelium results from stromal-epithelial interactions within the PT or if it represents cancerisation of a PT by carcinoma arising in immediately adjacent mammary tissue [8].

The prevalence of malignant epithelial transformation in PT of 6% observed in the current population-based series is higher compared to the <1% prevalence reported by Tan, et al. at the Singapore General Hospital, Singapore [2]. As there are few studies that have documented the prevalence of this rare combination of pathologies with which the current study’s findings may be compared, this difference must be interpreted with caution. A lower incidence of breast cancer in Singapore (65 per 100,000) [13] compared to the province British Columbia, Canada (100 per 100,000) [14] could be a possible factor contributing to the observed variations. However, this hypothesis is speculative since the etiology of carcinoma within PT has not yet been elucidated. In other words, carcinoma within PT of the breast could be a separate entity and thus may have a different pathogenesis compared to carcinoma of the breast without PT.

Carcinoma in PT has been described in all subtypes of PT, but until 2013 breast carcinoma was rarely reported in borderline PT [4, 7, 15-17]. However, similar to a recent case series by Sin, et al. [9] the majority of our cases had borderline PT, and it thus seems that a coexisting carcinoma occurs irrespectively of PT subtype. Studies with larger sample sizes are needed to investigate possible relationships between coexisting carcinoma and histologic features of PT. However, because of the rarity of the disease, analysis of pooled data might be the preferred method, provided that detailed histological data are available.

In the majority of our cases, carcinoma within a PT was detected at pathologic examination after definitive surgery. PTs tend to grow quickly and can advance to a large size. Therefore, thorough sampling is most essential in PT, not only to full examine the stromal component to classify subtype, but also to diagnose malignant epithelial transformation in the same lesion elsewhere [9].

Invasive carcinoma arising in PT is a rare phenomenon with fewer than 20 cases described in the literature [4, 7]. Our case series included one patient with a grade 1 IDC with extensive infiltration throughout a large PT without regional or distant metastasis. Based on these features and biomarker status, the patient underwent mastectomy and additional radiotherapy and hormonal therapy. After approximately 6.5 years, there was no evidence of disease recurrence.

In malignant PT, metastases and death are observed in 22% [1], underscoring the need for complete surgical resection in this subset of aggressive PTs [18]. Among the 40 cases of carcinoma within PT published in literature overviews and case series with available survival data [4, 8-10], four deaths have been reported [10, 19-21], of which two were due to an unrelated cause [10, 20]. The other two cases with carcinoma within malignant PT died of distant metastases, predominantly to bone and lungs [19, 21]. Evaluation of the stromal elements in the excisional biopsies allowed interpretation as metastases of the PT. Both patients did not have evidence of lymph node metastasis, as may be expected since axillary lymph node metastases in PT of the breast are rare [1].

There is a lack of standardization in the treatment of PT in general, including cases with malignant epithelial transformation which are even more rare [9]. As the presence of coexistent carcinoma impacts management decisions, a multidisciplinary approach with input from breast cancer surgeons, pathologists, medical oncologists and radiation oncologists is needed to individualize management, including considerations for axillary nodal staging, careful pathologic examination to establish pathologic stage, margin status and biomarkers to tailor adjuvant systemic and local therapy decision-making [8, 22].

The limitations of the current study include its retrospective design subject to inherent selection biases, small samples due to disease rarity, and relatively short follow-up time. In a study from the same institution of 183 cases of PT with a median follow-up time of 65 months, local recurrence was observed in 8.7%, distant metastases in 4.4%, and cause-specific deaths in 3.8% [23]. Among the 11 cases examined in the current analysis, no recurrence or cancer-specific deaths were observed at a mean follow-up of 54 months, suggesting that recurrence and survival outcomes were not inferior relative to PT without malignant epithelial transformation. However, as invasive breast cancer may recur years after diagnosis and treatment, longer follow-up is needed. Acknowledging these limitations, the study contributes population-based data documenting the prevalence of malignant epithelial transformation in PT and offers data on treatment and short-term outcomes in this rare disease.

## Conclusions

In conclusion, we have documented a higher prevalence of malignant epithelial transformation in PT than reported in previous literature, and outcomes of our cases appeared favourable despite the presence of either in situ or invasive carcinoma within PT. Longer follow-up and larger samples, likely from pooled data, are needed to continue to advance our insight into this unique breast disease.
